# Neuropsychobiological Fingerprints of Chronic Fatigue in Sarcoidosis

**DOI:** 10.3389/fnbeh.2021.633005

**Published:** 2021-07-26

**Authors:** Sarah Kettenbach, Sina Radke, Tobias Müller, Ute Habel, Michael Dreher

**Affiliations:** ^1^Department of Pneumology and Intensive Care Medicine, RWTH Aachen University Hospital, Aachen, Germany; ^2^Department of Psychiatry, Psychotherapy and Psychosomatics, Medical Faculty, RWTH Aachen University, Aachen, Germany

**Keywords:** sarcoidosis, rare lung diseases, chronic fatigue, functional magnetic resonance imaging, angular gyrus

## Abstract

**Background:**

Chronic fatigue is a prominent symptom in many sarcoidosis patients, affecting quality of life and interfering with treatment. This study investigated neuropsychobiological mechanisms and markers of chronic fatigue in sarcoidosis.

**Methods:**

Thirty patients with a histological diagnosis of sarcoidosis were included. The Multidimensional Fatigue Inventory was used to define patients with and without chronic fatigue. All patients were then characterised using several depression, quality of life questionnaires, and executive functioning. Cognitive functioning and underlying neural correlates were assessed using an n-back task measuring working memory and (sustained) attention during functional magnetic resonance imaging. Sarcoidosis disease activity was determined using lung function, laboratory parameters, and exercise capacity.

**Results:**

Nineteen patients had chronic fatigue and 11 did not; both groups had similar demographic and disease activity characteristics. Chronic fatigue patients showed more symptoms of depression and anxiety, and lower quality of life. During the n-back task, chronic fatigue was associated with a smaller increase in brain activation with increasing task difficulty versus the group without fatigue, especially in the angular gyrus.

**Conclusion:**

Inadequate adjustment of brain activation with increasing demands appears to be a potential neurobiological marker of chronic fatigue in sarcoidosis patients. The angular gyrus, which plays an important role in the working memory system, was the major area in which fatigue patients showed smaller increase of brain activation compared to those without fatigue. These findings might be relevant for a deeper understanding of chronic fatigue mechanisms in sarcoidosis and future clinical treatment of this disabling syndrome.

**Trial Registration:**

ClinicalTrials.gov, Trial registration number: NCT04178239

Date of registration: November 26, 2019, retrospectively registered

URL: https://clinicaltrials.gov/ct2/show/NCT04178239.

## Introduction

Sarcoidosis is a systemic granulomatous disease of unknown aetiology characterised by non-caseating epithelioid cell granulomas. Clinical manifestations and symptoms are highly variable (Marcellis et al., 2011; Drent et al., 2012; Lal et al., 2015). Sarcoidosis patients frequently experience chronic fatigue (CF), characterised by severe disabling fatigue, physical and/or mental weariness, reduced sensation of strength, lack of energy, feeling of complete exhaustion, myalgia, sleep reversal, and/or low spiritedness without any organic cause (Korenromp et al., 2011; Marcellis et al., 2011). The prevalence of CF in sarcoidosis varies from 50 to 80%; its aetiology is not yet understood (Korenromp et al., 2011; Drent et al., 2012; Lal et al., 2015). CF markedly impairs quality of life (QoL), without any established possibility for adequate treatment (Kleijn et al., 2011; Drent et al., 2012; Fleischer et al., 2014; Lal et al., 2015). However, markers indicative of the occurrence, severity, or impact of CF in sarcoidosis have not yet been identified.

The aim of this study was to characterise CF in sarcoidosis based on its neuropsychobiological correlates, specifically whether CF in sarcoidosis leads to cognitive impairment and quantifiable neural alterations in the brain, and whether these features could be used to distinguish between sarcoidosis patients with versus without CF. To achieve this, we used a wide range of neuropsychological questionnaires and a common, standardised cognitive task (n-back) during functional magnetic resonance imaging (fMRI). The n-back task assesses attention and working memory (WM) (Caseras et al., 2006; Cook et al., 2007) and robustly reveals activations in a fronto-parieto-cerebellar network (Seghier, 2013; Jacola et al., 2014). CF would be expected to be associated with symptoms of depression and anxiety, and impaired QoL in sarcoidosis patients. An additional hypothesis was that sarcoidosis patients with CF would demonstrate alterations in activation of the fronto-parieto-cerebellar network during n-back task due to alterations in WM, similar to previous studies of CF patients without sarcoidosis (Caseras et al., 2006; Bartes-Serrallonga et al., 2014; Provenzano et al., 2020), and that reduced cognitive function would occur in parallel with reduced brain network activation as WM demands increase.

## Materials and Methods

### Study Subjects

Adult patients with histologically diagnosed sarcoidosis were screened for eligibility. Main exclusion criteria were MRI-related contraindications, such as past thoracic or ophthalmologic surgery, medical history of epilepsy, tinnitus or seizure, extensive tattoos, or current pregnancy. Eligible patients were divided into two groups (CF and no CF [NCF]) based on the Multidimensional Fatigue Inventory (MFI).

### Assessments

The MFI is an instrument to assess fatigue severity and has previously been used in studies of CF in sarcoidosis (Fleischer et al., 2014; Bosse-Henck et al., 2015). For each item, a score from 1 to 5 is possible, resulting in a total score of 20–100 (Smets et al., 1995). In this study, CF was diagnosed in patients with a total score of >53 based on the 75th percentile of norm values (Schwarz et al., 2003) as primary developed by Kuhnt et al. (2009) and used in several studies with cohorts of sarcoidosis patients (Hinz et al., 2011; Fleischer et al., 2014; Bosse-Henck et al., 2015).

In addition to the MFI, participants also completed the Fatigue Assessment Scale, which is frequently used in groups of sarcoidosis patients (Kleijn et al., 2011; Drent et al., 2012), and the Fatigue Impact Scale to assess the impact of fatigue on health-related QoL (Häuser et al., 2003).

Symptoms of depression were measured using two self-assessments (Allgemeine Depressionsskala (Hautzinger et al., 2012) and Rasch-basiertes Depressionsscreening (Forkmann et al., 2011)) and one clinical assessment (Hamilton Depression Scale (Hamilton, 1960)). Anxiety (State-Trait Anxiety Inventory (Spielberger et al., 1983)), QoL (WHOQOL-BREF (Harper, 1998)), subjective impairment of physical and psychological symptoms (Brief Symptom Inventory (Franke, 2000)), and executive functions (Trail Making Test (Reitan, 1956)) were also assessed.

Demographic data and disease history were recorded for all patients, and blood samples were taken. Standard clinical parameters for sarcoidosis disease activity, including complete blood count, circulating levels of C-reactive protein, soluble interleukin-2 receptor, neopterin, angiotensin-converting enzyme (ACE) polymorphism, and calcium were measured in serum and whole blood.

Whole body plethysmography (MasterLab, Viasys, Hoechberg, Germany) was performed according to current recommendations (Miller et al., 2005; Wanger et al., 2005). Norm values were calculated according to Matthys et al. (1995). Samples for arterial blood gas analyses were taken from patients’ arterialised earlobe while breathing room air (ABL 800 flex, Radiometer, Copenhagen, Denmark).

Exercise capacity was assessed using a standardised 6-min walking test (6MWT) (Ats Committee on Proficiency Standard for Clinical Pulmonary Function Laboratories, 2002). Oxygen saturation before and at the end of the 6MWT was assessed *via* pulsoximetry and dyspnoea was rated on the Borg Dyspnea Scale (Borg, 1982).

Mean differences in between the CF and NCF groups were tested using independent samples *t*-tests.

### n-Back Task

**FIGURE 1:**
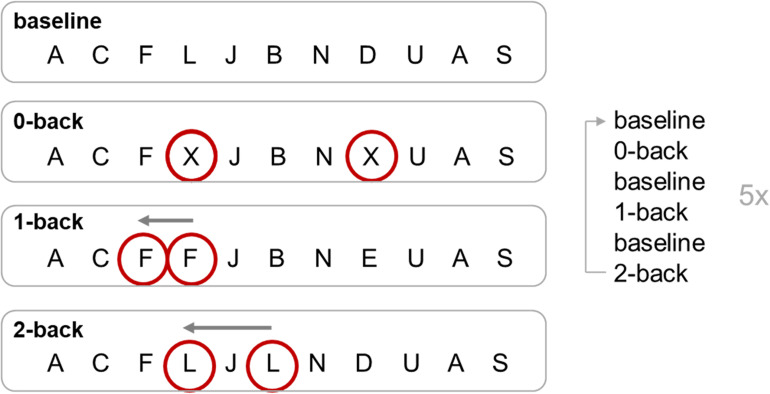
n-back task. Targets of each condition of the n-back task are circled. The 0-back, 1-back, and 2-back conditions were repeated five times, alternating with the baseline condition. For 0-back, a response on a certain target letter is required (button press for X); for 1-back, the response is required for a repetition of a target letter (e.g., second F); for 2-back, the response has to be on the last but one repetition of the target letter (e.g., L, J, L; response to the L).

During fMRI, a visual n-back task was performed, which included four conditions for parametric modulation of WM load. As a baseline condition (“fixation”), participants were presented with a series of letters that they should watch without any need to respond. In the 0-back condition, subjects were required to respond to the target letter “X” *via* button-press with their right index finger, and in the 1-back condition, they had to respond to the second of two consecutive identical letters. In the 2-back condition, participants had to respond to letters identical to the one presented two letters before (Figure 1). The target letter “X” from the 0-back task did not appear in the 1-back or 2-back task. Each of the three conditions 0-back, 1-back, and 2-back appeared five times, alternating with 15 baseline blocks. Every block was initiated by a 2.5-s task instruction, and stimuli were presented for 500 ms each. For each n-back block, 19 letters were presented, resulting in a block duration (including instructions) of 29.7 s. Baseline blocks included 10 stimuli, resulting in a block duration (including instructions) of 17.2 s. Total duration of the paradigm was 11 min 46 s. Stimuli were presented on a projection screen visible through a mirror attached to the head coil. Stimulus presentation and response acquisition were controlled *via* Presentation software (Neurobehavioral Systems, Inc., Albany, CA, United States). Average hit rates (number of correct responses divided by the total number of possible correct responses) and reaction times (time from presentation of the target until button-press) were measured for each condition for each participant.

Behavioural and cerebral responses on the n-back task for each subject were averaged per condition. For both behavioural and brain data, ANOVAs with condition ([baseline], 0-back, 1-back, and 2-back) as a within-subject factor and group (CF, NCF) as a between-subject factor were performed. Although hit rates were not normally distributed, ANOVAs have been found to be robust to distributional differences, even for small samples (Blanca et al., 2017).

Significance level was set to α = 0.05. Bonferroni and familywise error corrections were applied in IBM SPSS Statistics 24 and SPM12, respectively.

### Functional Magnetic Resonance Imaging

fMRI data were obtained on a 3-T Prisma MR scanner (Siemens Medical Systems, Germany). Images were obtained with a gradient-echo EPI sequence (TR 2500 ms, TE 30 ms, 3.1 × 3.1 × 3.1 mm^3^ voxel size, FoV 200 × 200 mm^2^, 38 slices, 77° flip angle, distance factor 25%). Additionally, a high-resolution structural image (3-D Magnetization Prepared Rapid Gradient Echo [MP-RAGE]) was acquired with the following parameters: TR 2000 ms, TE 3.03 ms, TI 900 ms, 9° flip angle, FoV = 256 × 256 mm^2^, 176 slices, 1 × 1 × 1 mm^3^ voxel size.

### fMRI Data Processing and Analysis

Statistical parametric mapping (SPM12, Welcome Department of Imaging Neuroscience, London) was used for pre-processing, and analyses with standard algorithms and parameters were applied unless otherwise specified. Pre-processing included realignment of data to correct for head movement, and slice timing. The mean functional image was coregistered and normalised to the Montreal Neurological Institute (MNI) single-subject template (Collins et al., 1994) using linear proportions and a non-linear sampling as derived from a segmentation algorithm (Ashburner, 2005). Images were spatially smoothed using an 8-mm full-width-at-half maximum Gaussian kernel.

In the GLM analysis for each subject, the four experimental conditions (baseline, 0-back, 1-back, and 2-back) were modelled block-wise by convolving vectors of onset times with the canonical haemodynamic response function. In addition, six head movement parameters from the realignment were included as regressors of no interest in the first-level model. Finally, images were high-pass filtered at 128 s, and an autoregressive AR(1) model was used to account for serial correlations in fMRI time series.

Statistical analyses at the group level were performed by subjecting the four task-relevant contrast images to a full factorial ANOVA with the factors condition (baseline, 0-back, 1-back, and 2-back) and group (CF and NCF). The main effects of condition and group, and interactions between these two factors, were tested. All effects were thresholded at *p* < 0.05 at cluster-level, familywise error corrected for multiple comparisons (pFWE < 0.05), with an underlying voxel-level threshold of *p* < 0.001, uncorrected. This entails a minimum cluster size of *k* > 217. Activation maxima are reported as Montreal Neurological Institute (MNI) coordinates; anatomical locations are based on SPM Anatomy Toolbox Version 2.2b (Eickhoff et al., 2005).

## Results

**FIGURE 2:**
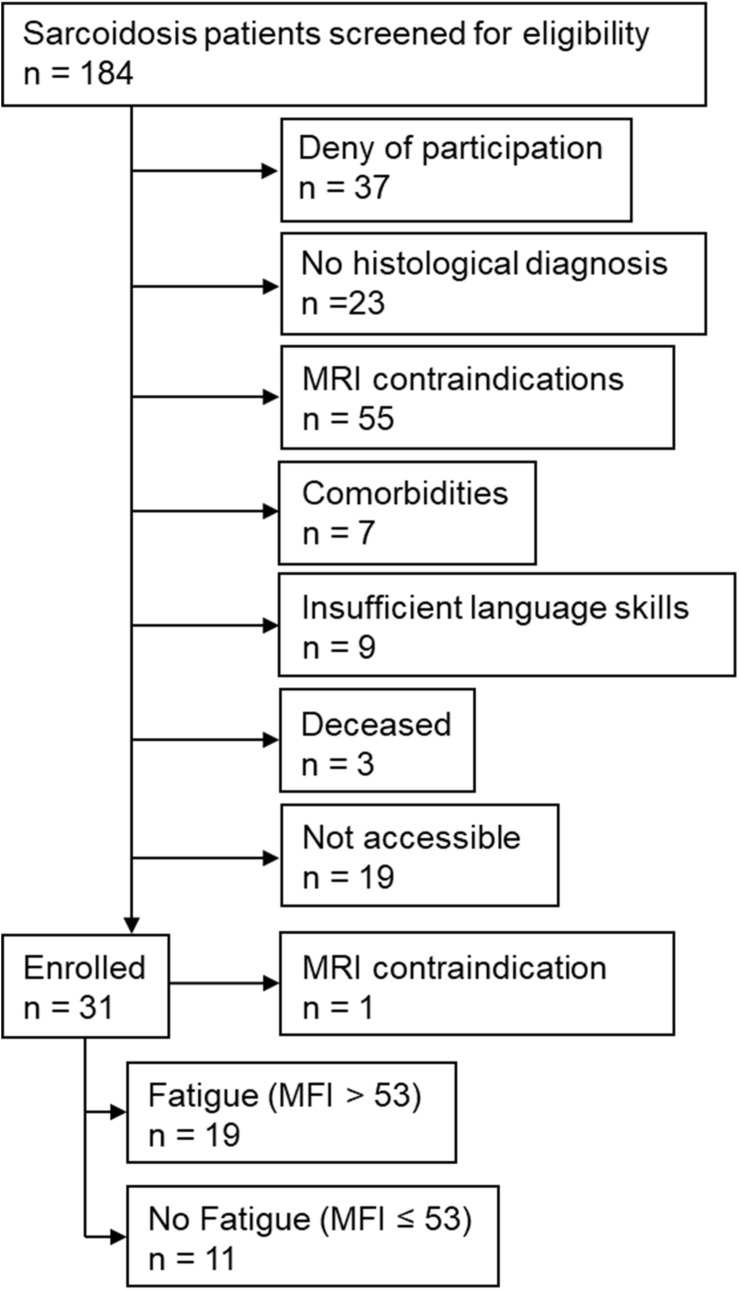
Study flow chart. MFI: Multidimensional Fatigue Inventory; MRI: magnetic resonance imaging.

Of 184 patients screened for eligibility, 30 patients were included into the study (Figure 2). Of these, 19 had an MFI score of >53 points and were therefore assigned to the CF group; the remaining 11 were assigned to the NCF group. The two groups did not differ significantly in age, height, weight, body mass index, duration of illness, number of affected organs, or laboratory parameters (Table 1). Lung function and 6MWT were also similar in the two groups (Table 2).

**TABLE 1:** Demographic data and laboratory values at baseline for sarcoidosis patients with and without chronic fatigue.

	CF (*n* = 19)	NCF (*n* = 11)	Between-group difference [95% CI]	*p*-value
**Demographic data**				
Age, years	46.4 ± 9.3	49.5 ± 10.1	−3.0 [−10.5, 4.4]	0.412
Height, cm	173.5 ± 8.8	174.8 ± 7.8	−1.3 [−7.9, 5.2]	0.679
Weight, kg	79.6 ± 18.1	78.4 ± 14.8	1.2 [−12.0, 14,4]	0.852
Body mass index, kg/m^2^	26.2 ± 4.7	25.5 ± 3.9	0.7 [−2.7, 4.2]	0.667
Time since diagnosis, months	79.9 ± 65.5	62.0 ± 41.8	17.9 [−27.2, 63.1]	0.422
Number of affected organs, n	2.4 ± 0.9	2.4 ± 0.8	−0.03 [−0.7, 0.6]	0.920
**Laboratory parameters**				
Leucocytes, /nl	7.00 ± 2.24	6.34 ± 2.12	0.7 [−1.0, 2.4]	0.429
Erythrocytes, /pl	4.97 ± 0.49	4.97 ± 0.53	0.001 [−0.4, 0.4]	0.996
Haemoglobin, g/dL	15.00 ± 1.26	14.85 ± 1.26	0.1 [−0.8, 1.1]	0.757
Haematocrit, %	44.13 ± 3.44	43.66 ± 3.42	0.5 [−2.2, 3.1]	0.722
Thrombocytes, /nl	270.05 ± 58.39	239.09 ± 22.73	31.0 [−6.9, 68.8]	0.050
Lymphocytes, %	21.67 ± 8.32	24.35 ± 8.91	−2.7 [−9.4, 4.0]	0.421
Eosinophilic leucocytes, %	2.96 ± 2.76	2.41 ± 1.71	0.5 [−1.4, 2.5]	0.562
Calcium, mmol/L	2.39 ± 0.12	2.43 ± 0.08	−0.05 [−0.13, 0.04]	0.266
C-reactive protein, mg/L	3.48 ± 3.96	2.21 ± 1.74	1.3 [−1.3, 3.9]	0.235
sIL-2 receptor, U/ml	564.16 ± 421.14	673.73 ± 526.33	−109.6 [−467.7, 248.6]	0.536
Neopterin, nmol/L	10.34 ± 9.90	10.84 ± 7.55	−0.5 [−7.9, 6.9]	0.891
ACE-polymorphism, mU/L	28.96 ± 16.49	24.51 ± 24.18	4.5 [−10.7, 19.7]	0.553

*Data are presented as mean ± standard deviation. ACE: angiotensin-converting enzyme; CF: chronic fatigue; NCF: no chronic fatigue; sIL-2: soluble interleukin-2.*

**TABLE 2:** Lung function parameters and 6-min walking test results.

	CF (*n* = 19)	NCF (*n* = 11)	Between-group difference [95% CI]	*p*-value
**Lung function**				
TLC, % predicted	105.789 ± 8.70	100.23 ± 13.49	5.6 [−2.7, 13.8]	0.429
VC, % predicted	105.57 ± 9.43	100.19 ± 14.47	5.4 [−3.5, 14.3]	0.996
RV, % predicted	109.44 ± 16.71	107.67 ± 27.41	1.8 [−14.7, 18.2]	0.757
FEV_1_, % predicted	99.39 ± 25.33	95.92 ± 20.90	3.5 [−15.0, 22.0]	0.722
FEV_1_/VC IN, %	80.21 ± 7.79	76.06 ± 10.38	4.1 [−2.7, 11.0]	0.050
R tot, % predicted	106.22 ± 36.44	107.56 ± 37.3	−1.3 [−29.9, 27.2]	0.421
DLCOc SB, % predicted	76.68 ± 12.04	69.74 ± 16.13	6.9 [−4.8, 18.7]	0.562
RV SB, % predicted	96.55 ± 17.06	85.32 ± 18.75	11.2 [−4.0, 26.5]	0.266
DLCOc/Va, % predicted	83.05 ± 13.02	80.26 ± 12.10	2.8 [−8.2, 13.7]	0.235
PaO_2_, mmHg	75.68 ± 7.26	75.06 ± 9.90	0.6 [−5.8, 7.1]	0.536
PaCO_2_, mmHg	35.71 ± 3.61	36.12 ± 4.15	−0.4 [−3.4, 2.5]	0.891
pH	7.44 ± 0.03	7.43 ± 0.02	0.01 [−0.01, 0.03]	0.553
**6-min walking test**				
Distance, m	544 ± 54	554 ± 62	−9.2 [−54.0, 35.6]	0.677
SpO_2_ after exercise, %	98.5 ± 1.4	99.2 ± 1.3	−0.7 [−1.7, 0.4]	0.193
Difference of SpO_2_, %	0.2 ± 1.4	0.2 ± 0.6	−0.02 [−0.92, 0.89]	0.973
BDS score after exercise	2.1 ± 1.8	1.5 ± 1.8	0.7 [−0.8, 2.1]	0.348
Change in BDS score	1.9 ± 1.5	1.4 ± 1.6	0.6 [−0.6, 1.8]	0.338

*Data are presented as mean ± standard deviation. BDS: Borg Dyspnea Scale; CI: confidence interval; DLCOc SB: diffusing capacity for carbon monoxide single breath; DLCOc/Va: diffusing capacity for carbon monoxide per ventilated volume; FEV_1_: forced expiratory volume at 1 s; FEV_1_/VC IN: Tiffenau–Index; PaCO_2_: arterial partial pressure of carbon dioxide; PaO_2_: arterial partial pressure of oxygen; RV: residual volume; R tot: total resistance; RV SB: residual volume single breath; SpO_2_: oxygen saturation; TLC: total lung capacity; VC: vital capacity.*

### Questionnaires

Fatigue, depression, and anxiety questionnaires’ overall and sub-domain scores showed significantly greater fatigue, anxiety, and depression in the CF versus NCF group (all *p* < 0.001). WHOQOL-BREF physical health and global domains differed significantly between the two groups; after correction for multiple testing, no significant differences were found in the social relationships, psychologic health, and environmental health domains. In addition, the CF group showed more subjective impairments in physical and psychological symptoms based on the BSI, both overall and in the somatisation, obsessive–compulsive, interpersonal sensitivity, depression, anxiety, hostility, phobia, and extra items domains (all *p* < 0.05); there were no statistically significant between-group differences in the paranoia (*p* = 0.05) and psychoticism (*p* = 0.149) domains. Executive functions were similar in the CF and NCF groups (all Table 3).

**TABLE 3:** Fatigue, depression, quality of life, and executive functioning in sarcoidosis patients with versus without chronic fatigue.

	CF (*n* = 19)	NCF (*n* = 11)	Between-group difference [95%CI]	*p*-value	*d*_*Cohen*_
MFI total	66.9 ± 10.0	39.5 ± 9.4	27.4 [19.8, 35.0]	<0.001*	−2.76
MFI general fatigue	14.6 ± 2.7	8.3 ± 2.9	6.4 [4.2, 8.5]	<0.001*	−2.27
MFI physical fatigue	14.5 ± 2.1	8.8 ± 3.3	5.7 [3.7, 7.6]	<0.001*	−2.32
MFI red. activity	13.7 ± 2.8	8.4 ± 2.2	5.4 [3.4, 7.4]	<0.001*	−2.04
MFI red. motivation	11.2 ± 3.0	7.1 ± 2.5	4.1 [1.9, 6.3]	0.001*	−1.45
MFI mental fatigue	12.9 ± 3.2	7.0 ± 3.0	5.9 [3.5, 8.3]	<0.001*	−1.89
FAS total	32.1 ± 6.1	18.5 ± 5.3	13.7 [9.1, 18.2]	<0.001*	−2.33
FIS total	80.0 ± 23.7	25.3 ± 21.7	54.7 [36.9, 72.6]	<0.001*	−2.38
ADS	17.6 ± 9.8	4.5 ± 4.0	13.1 [6.8, 19.5]	<0.001*	−1.6
DESC-I	7.5 ± 7.3	0.6 ± 1.0	6.9 [2.3, 11.5]	0.001*	−1.17
DESC-II	8.4 ± 7.4	1.6 ± 3.6	6.8 [1.9, 11.7]	0.009	−1.08
HAMD	4.1 ± 2.1	2.7 ± 0.8	1.4 [0.2, 2.6]	0.029	−0.8
STAI-X1	38.3 ± 9.0	29.3 ± 6.0	9.0 [2.8, 15.3]	0.006	−1.12
STAI-X2	44.6 ± 10.3	30.3 ± 6.9	14.3 [7.1, 21.5]	<0.001*	−1.55
WHOQOL-BREF global domain	45.4 ± 20.9	76.1 ± 14.2	−30.7 [−43.9, −17.5]	<0.001*	1.63
WHOQOL-BREF physical health domain	49.4 ± 22.2	81.2 ± 10.5	−31.7 [−44.0, −19.4]	<0.001*	1.69
WHOQOL-BREF psychologic health domain	63.4 ± 18.9	78.8 ± 7.1	−15.4 [−25.3, −5.5]	0.004	0.979
WHOQOL-BREF social relationships domain	67.5 ± 20.6	78.0 ± 15.0	−10.5 [−25.1, 4.1]	0.152	0.56
WHOQOL-BREF environmental health domain	74.0 ± 13.6	83.2 ± 10.7	−9.2 [−19.0, 0.6]	0.064	0.73
BSI total	0.72 ± 0.52	0.19 ± 0.17	−0.5 [−0.8, −0.3]	<0.001*	−1.24
TMT-A	30.34 ± 16.0	26.5 ± 10.6	3.8 [−7.3, 14.9]	0.486	0.268
TMT-B	63.3 ± 32.1	62.3 ± 25.1	1.0 [−22.1, 24.2]	0.927	−0.035
Difference TMT-B minus -A	32,9 ± 26,8	35,7 ± 27,0	−2,7 [−23.6, 18,1]	0.787	−0.103

Data are presented as mean ± standard deviation. Significant results after Bonferroni correction are marked with *. Magnitude of effect size is given by d_Cohen._ ADS: Allgemeine Depressionsskala (General Depression Scale); BSI: Brief Symptom Inventory; DESC: Rasch-basiertes Depressionsscreening (Depression Screening); FAS: Fatigue Assessment Scale; FIS: Fatigue Impact Scale; HAMD: Hamilton Depression Scale; STAI: State-Trait Anxiety Inventory; TMT: Trail Making Test; WHOQOL-BREF: World Health Organization quality of life assessment short form.

### n-Back Task

**FIGURE 3:**
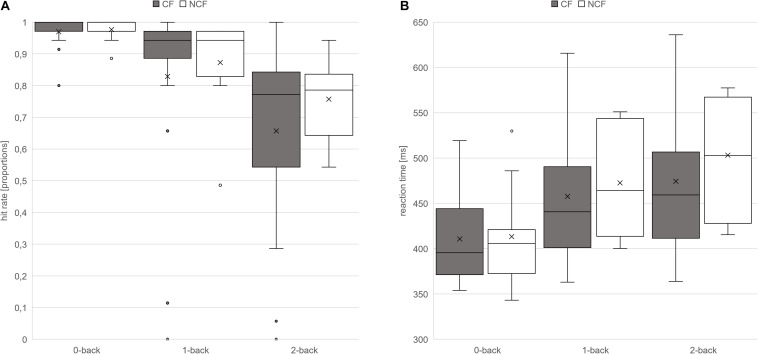
Hit rates and response times in n-back task. Hit rates (in proportions) **(A)** and response times (in milliseconds) **(B)** on the n-back task in sarcoidosis patients with versus without chronic fatigue presented as box–whisker plots. Horizontal lines denote (from bottom to top) minimum, third quartile, median, first quartile, and maximum. Crosses indicate arithmetic means; points denote statistical outliers.

Hit rates in the n-back task decreased with increasing task difficulty in both groups, and reaction times increased. There was a significant main effect of condition (within-subject effect), but not group (between-subject effect), for both hit rates and reaction times, with no significant interaction (Table 4 and Figure 3).

**TABLE 4:** Behavioural data n-back task.

	CF (*n* = 19)	NCF (*n* = 11)	*p*-value	Partial eta-squared
**Working memory condition**				
Hit rates, proportion				
0-back	0.96 ± 0.05	0.98 ± 0.04		
1-back	0.83 ± 0.28	0.87 ± 0.14		
2-back	0.66 ± 0.30	0.76 ± 0.13		
Reaction times, ms				
0-back	411 ± 52	413 ± 53		
1-back	458 ± 75	473 ± 59		
2-back	474 ± 80	503 ± 64		
**Hit rates, proportion**				
Group (between-subject effect)			0.325	0.036
Condition (within-subject effect)			<0.001	0.435
0-back–1-back			0.071	
0-back–2-back			<0.001	
1-back–2-back			<0.001	
Interaction			0.483	0.027
**Reaction times, ms**				
Group (between-subject effect)			0.330	0.038
Condition (within-subject effect)			<0.001	0.468
0-back–1-back			<0.001	
0-back–2-back			<0.001	
1-back–2-back			0.185	
Interaction			0.546	0.024

*Working memory condition data are presented as mean ± standard deviation. Partial eta-squared depicts magnitude of effect size.*

### fMRI Data

**FIGURE 4:**
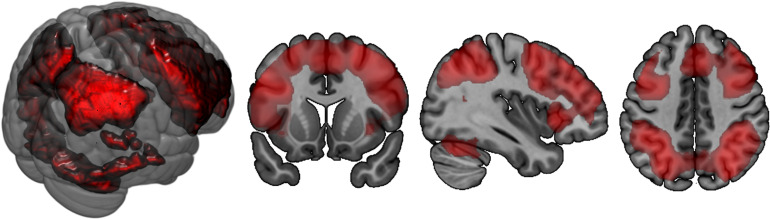
Brain activation corresponding to the main effect of condition in n-back task: 2-back > 0-back. Highlighted areas are those that demonstrate more activation in the cognitively more challenging 2-back condition versus the cognitively less challenging 0-back condition.

In both groups, more difficult conditions of the n-back task elicited more brain activation versus conditions that required less WM, especially in parietal and frontal brain areas (Table 5 and Figure 4). Simultaneously, middle cingulate cortex, rolandic operculum, insular lobe, and superior medial gyrus showed decreasing activation in the 2-back versus less demanding conditions. No significant between-subject effect was found.

**TABLE 5:** Brain activation corresponding to the main effect of condition in n-back tasks (whole-brain condition effects, all *p* < 0.05 [familywise error rate (FWE)-corrected]; only the maximum peak in grey matter is reported for each cluster).

Region	Number of active voxels in the cluster (*k*)	Side	MNI			*t*-value
			*x*	*y*	*z*	
**2-back > 1-back**						
Inferior frontal gyrus (pars triangularis)	7685	L	−38	16	30	6.74
Superior parietal lobule	6073	L	−26	−66	54	6.61
Middle frontal gyrus	1012	R	38	38	40	4.76
Cerebellum (VI)	834	R	12	−72	−26	5.81
Middle frontal gyrus	265	L	−28	48	20	5.00
Insular lobe	257	R	32	26	0	4.97
**2-back > 0-back**						
Middle frontal gyrus	21401	L	−26	2	56	9.87
Superior parietal lobule	12119	L	−28	−62	44	10.35
Cerebellum (VI)	6349	R	10	−78	−22	9.34
Thalamus	944	R	12	−2	2	4.90
Middle temporal gyrus	257	R	46	−54	12	3.98
**2-back > baseline**						
Posterior medial frontal	32824	R	4	24	48	11.15
Inferior parietal lobule	27229	L	−42	−44	48	9.81
**1-back > 0-back**						
Superior parietal lobule	19251	R	32	−68	50	8.04
Inferior frontal gyrus (pars triangularis)	11731	R	50	28	24	8.31
Inferior frontal gyrus (pars triangularis)	5831	L	−44	34	26	7.08
Thalamus	1176	R	20	−20	12	5.26
Middle cingulate cortex	465	L	−2	−28	30	5.16
Caudate nucleus	269	L	−10	−2	16	4.42
**1-back > baseline**						
Posterior medial frontal	78791	R	6	8	56	10.56
**0-back > baseline**						
Posterior medial frontal	36244	L	−4	0	50	9.49
Cerebellum (VI)	418	L	−38	−52	−28	5.64
**1-back > 2-back**						
Middle cingulate cortex	1285	L	−12	−28	50	5.01
Rolandic operculum	1193	L	−40	−18	18	6.04
Insular lobe	898	R	38	−14	8	4.59
Superior medial gyrus	889	L	−10	58	24	5.02
Postcentral gyrus	267	L	−34	−26	48	3.73
**0-back > 2-back**						
Superior medial gyrus	940	L	−10	58	26	5.39
Rolandic operculum	501	R	40	−20	22	4.25
Rolandic operculum	356	L	−40	−14	16	4.47
**baseline > 2-back**						
Precuneus	407	L	−6	−52	20	4.54
Superior medial gyrus	292	L	−2	60	2	4.07

*L: left; MNI: Montreal Neurological Institute; R: right.*

**FIGURE 5:**
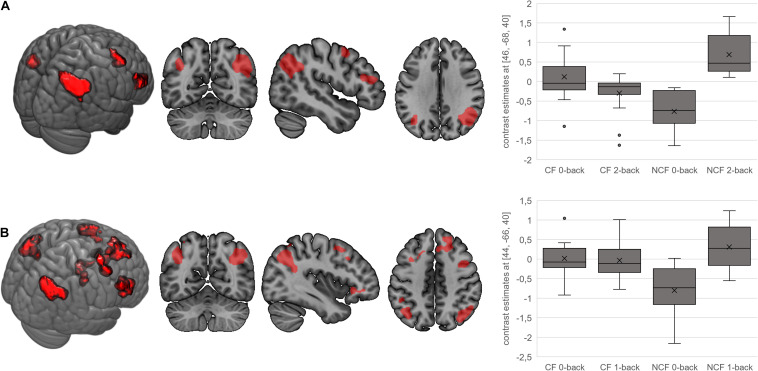
Interactions for fatigue > no fatigue in n-back task. Highlighted areas show brain regions that show more activation in the chronic fatigue (CF) versus no chronic fatigue (NCF) group in the contrasts 0-back > 2-back **(A)** and 0-back > 1-back **(B)**. Box–whisker plots show brain activation of the major cluster’s maximum voxel in arbitrary units. Horizontal lines denote (from bottom to top) minimum, third quartile, median, first quartile, and maximum. Crosses indicate arithmetic means; points denote statistical outliers.

In terms of interactions, the CF group showed more angular gyrus activation than the NCF group in the contrast 0-back > baseline condition. All other significant interactions showed less activation with increasing n-back task difficulty in the CF versus NCF group (Table 6 and Figure 5). Affected brain areas were especially the angular gyrus and, furthermore, the inferior frontal gyrus, middle frontal gyrus, superior medial gyrus, thalamus, anterior cingulate cortex, and superior parietal lobule. Exploratory correlational analyses of questionnaire, clinical, behavioural, and fMRI data yielded no significant results after correction for multiple testing. Correction for covariates gender and age in significant interactions between group and n-back contrast revealed no significant changes in results.

**TABLE 6:** Interactions CF > NCF in n-back tasks between the two groups (chronic fatigue, no chronic fatigue) and the four different conditions of the n-back task (all *p* < 0.05 [familywise error rate (FWE)]-corrected; only the maximum peak in grey matter is reported for each cluster).

Region	Number of active voxels in the cluster (*k*)	Side	MNI			*t*-value
			*x*	*y*	*z*	
**0-back > baseline**						
Angular gyrus	324	L	−54	−66	24	4.27
**0-back > 2-back**						
Angular gyrus	1331	R	46	−68	40	6.81
Inferior frontal gyrus (pars triangularis)	377	R	50	38	16	4.50
Middle frontal gyrus	266	R	40	8	46	4.40
Angular gyrus	235	L	−38	−62	34	4.54
**0-back > 1-back**						
Angular gyrus	913	R	44	−66	40	5.37
Angular gyrus	695	L	−38	−68	40	5.22
Inferior frontal gyrus (pars triangularis)	613	R	50	28	22	5.27
Superior medial gyrus	594	R	16	34	58	4.32
Middle frontal gyrus	441	L	−36	8	52	4.88
Thalamus	330	R	18	−12	16	4.19
Inferior frontal gyrus (pars orbitalis)	298	L	−46	30	−10	5.35
Middle frontal gyrus	286	R	36	2	62	4.57
Anterior cingulate cortex	252	L	−6	34	24	4.26
**baseline > 2-back**						
Angular gyrus	739	R	38	−74	46	5.43
**baseline > 1-back**						
Superior parietal lobule	519	R	34	−72	50	4.53
Inferior frontal gyrus (pars triangularis)	335	R	50	28	22	4.40

*L: left; MNI: Montreal Neurological Institute; R: right.*

## Discussion

This is the first time that CF in sarcoidosis has been characterised neuropsychobiologically using fMRI and is therefore an important step towards more comprehensive investigation of this poorly understood and disabling symptom. This study has successfully shown that in addition to being a subjective phenomenon, CF can also be characterised on a cerebral level.

CF patients, who demonstrated fatigue in all subdomains of the fatigue scales used, showed significantly less brain activation with increasingly complex cognitive conditions versus those without fatigue, during the n-back task. Disease activity was similar in both groups. Furthermore, CF patients demonstrated more symptoms of depression and anxiety, worse QoL, and more subjective impairment due to physical and psychological symptoms, similar to results of other studies in sarcoidosis cohorts (Kleijn et al., 2011; Korenromp et al., 2011; Drent et al., 2012; Lower et al., 2013; Saligan, 2014). These results underline the high impact of CF in sarcoidosis and the important need for further investigation of this debilitating symptom and potential treatment strategies.

The n-back task is widely used for assessment of (sustained) attention and WM (Bartes-Serrallonga et al., 2014; Jacola et al., 2014). Using this task, we could show alterations in the neural correlates underlying these functions in fatigued sarcoidosis patients. WM performance (hit rates and reaction times in the n-back task) did not significantly differ between patients with versus without fatigue, but fatigue patients tended to show fewer correct responses while differences between the two groups became greater as task difficulty increased. A similar pattern was found in a study of WM in CF syndrome (independent of any somatic disease) (Caseras et al., 2006). It is possible that the brain compensates for fatigue at lower levels of performance and that the effects of fatigue might only be detected at a higher cognitive demand, e.g., 3-back task, 4-back task. A compensation of fatigue at lower WM demand is revealed additionally in the form of reversed angular gyrus activity with progressively difficult testing (decrease in CF, increase in NCF group).

Patients with versus without fatigue showed more activation in the angular gyrus in the 0-back task compared with the baseline condition. For all other comparisons showing significant interactions, the CF group had less activation in more demanding tasks compared with the NCF group. Areas showing reduced activation during more demanding tasks in the CF group were the angular gyrus, inferior frontal gyrus, middle frontal gyrus, superior medial gyrus, thalamus, anterior cingulate cortex, and superior parietal lobule. These areas, which all show significant activation in the underlying contrasts as well, are mostly located in frontal and parietal regions, which are known to be part of the WM network (Caseras et al., 2006; Nissim et al., 2016). Activation patterns found in our study therefore illustrate an impact of CF on WM function and its underlying neural correlates in sarcoidosis patients.

Interactions in terms of CF patients showing a reduced activation with increasing task difficulty compared to the NCF group have been detected in a fMRI study regarding CF independent from sarcoidosis as well (Caseras et al., 2006). Main regions in this study are the right middle frontal gyrus as well as the bilateral parietal cortex; they are therefore partly overlapping with the clusters in our study. Our main cluster of activation in interactions, the angular gyrus, has so far proven to play a role in CF independent from sarcoidosis in terms of n-back interactions.

The angular gyrus, which occupies a posterior part of the inferior parietal lobule (Seghier, 2013), was the major cluster of activation in almost all significant interactions. In previous studies, the angular gyrus has shown the greatest change in global connectivity in the different conditions of the n-back task (Seghier, 2013; Vatansever et al., 2017). Therefore, it appears to be one of the major connector hubs linking different cerebral subsystems and to be responsible for reorienting the attentional system towards relevant information. It is also known to be an important part of the default mode network, which is actively involved in the n-back task and thus plays an important role in WM (Seghier, 2013; Vatansever et al., 2017). Previous studies on CF syndrome described less organised default mode connectivity as a potential diagnostic biomarker of CF (Shan et al., 2018). Along these lines, our findings suggest that CF patients do not recruit their WM system and functions in the bilateral angular gyrus according to the task demands in the same way as sarcoidosis patients without CF. They demonstrate less cognitive involvement and an inferior cerebral processing than NCF patients (depicted by their minor increase in activation with increased task difficulty). This is supported by the fact that default mode network connectivity (of which the angular gyrus forms an important part) in CF syndrome in the absence of sarcoidosis is less organised and could potentially be used as a diagnostic biomarker for CF (Shan et al., 2018).

Other clusters of activation in significant interactions are, among others, the middle frontal gyrus as well as the inferior frontal gyrus. These regions have also proven to be part of the WM system; the middle frontal gyrus plays a crucial role in regulating attention networks and is known to be involved in attention processing and sustained attention control (Dumas et al., 2013; Song et al., 2019; Salminen et al., 2020). The CF’s incapacity of recruiting these areas the same way NCF patients do once again underlines minor cognitive involvement and inferior cerebral processing in CF. This is supported by the fact that these regions that strike out significantly in interactions are part of the underlying contrasts. Middle frontal gyrus has as well been detected as a cluster of less activation with increasing n-back task demand in CF independent from sarcoidosis (Caseras et al., 2006). The similarity in results depicts a possible transferability between CF in the presence or absence of somatic diseases such as sarcoidosis. As the n-back task in fMRI has been considered to be a potential diagnostic biomarker in CFS independent from sarcoidosis (Caseras et al., 2006; Cook et al., 2007; Provenzano et al., 2020), it proves to be one for CF in sarcoidosis as well.

Our findings showing similar disease parameters and demographic data in sarcoidosis patients with versus without CF are similar to the majority of those in the published literature (Marcellis et al., 2011; Drent et al., 2012; Saligan, 2014). The lack of association between fatigue and clinical measurements highlights the relevance of our results. Differences between the CF and NCF groups in sarcoidosis cannot be explained by alterations in disease activity but can clearly be demonstrated using clinical questionnaires and assessment of cognitive functions (as visualised by fMRI).

However, our sample size is quite small, and MRI contraindications could have contributed to selection bias. Generalisability is limited by the absence of a healthy comparator population. Therefore, our findings (especially those relating to fMRI) need to be confirmed in a larger sample of sarcoidosis patients and healthy controls.

## Conclusion

In conclusion, we found a significant neuropsychobiological marker of fatigue in sarcoidosis patients with CF, who showed similar disease activity to sarcoidosis patients without CF. Those with CF appeared to differ in allocation of resources necessary for successful mastering of cognitive challenges, being unable to adequately recruit brain areas responsible for attention and WM in the same way that patients without fatigue do, particularly regarding the angular gyrus. This is consistent with findings in CF patients without sarcoidosis. Due to the high prevalence and detrimental impact of CF in sarcoidosis patients, our findings form an important basis for further investigations of biomarkers of fatigue and possible treatment strategies.

## Data Availability Statement

The raw data supporting the conclusion of this article will be made available by the authors, without undue reservation.

## Ethics Statement

The studies involving human participants were reviewed and approved by Institutional Review Board RWTH Aachen University (approval number EK 249/16). The patients/participants provided their written informed consent to participate in this study.

## Author Contributions

SK, TM, UH, and MD designed the study. SK, SR, UH, and MD analysed the data. SK performed the measurements and wrote the manuscript. SR, UH, and MD corrected the manuscript. All authors read and approved the final manuscript.

## Conflict of Interest

The authors declare that the research was conducted in the absence of any commercial or financial relationships that could be construed as a potential conflict of interest.

## Publisher’s Note

All claims expressed in this article are solely those of the authors and do not necessarily represent those of their affiliated organizations, or those of the publisher, the editors and the reviewers. Any product that may be evaluated in this article, or claim that may be made by its manufacturer, is not guaranteed or endorsed by the publisher.

## Abbreviations

CF: chronic fatigue
fMRI: functional magnetic resonance imaging
MFI: Multidimensional Fatigue Inventory
NCF: no chronic fatigue
QoL: quality of life
WM: working memory.
